# Loneliness in Posttraumatic Stress Disorder: A Neglected Factor in Accelerated Aging?

**DOI:** 10.3390/jal2040027

**Published:** 2022-12-09

**Authors:** Barton W. Palmer, Mariam A. Hussain, James B. Lohr

**Affiliations:** 1 Center of Excellence for Stress and Mental Health, Veterans Affairs San Diego Healthcare System, San Diego, CA 92161, USA; 2 Department of Psychiatry, University of California San Diego, La Jolla, CA 92037, USA; 3 Mental Illness Research, Education, and Clinical Center, Veterans Affairs San Diego Healthcare System, San Diego, CA 92161, USA; 4 Department of Neurosciences, University of California San Diego, La Jolla, CA 92161, USA

**Keywords:** aging, social isolation, stress disorders, post-traumatic, risk factors, comorbidity

## Abstract

Prior research suggests that people with Posttraumatic Stress Disorder (PTSD) may experience a form of accelerated biological aging. In other populations, loneliness has been shown to elevate risk for many of the same components of accelerated biological aging, and other deleterious outcomes, as seen in people with PTSD. Although standard diagnostic criteria for PTSD include “feelings of detachment or estrangement from others”, the relationship of such feelings to the concept of loneliness remains uncertain, in par potentially due to a failure to distinguish between loneliness versus objective social isolation. In order to catalyze wider research attention to loneliness in PTSD, and the potential contribution to accelerated biological aging, the present paper provides three components: (1) a conceptual overview of the relevant constructs and potential interrelationships, (2) a review of the limited extant empirical literature, and (3) suggested directions for future research. The existing empirical literature is too small to support many definitive conclusions, but there is evidence of an association between loneliness and symptoms of PTSD. The nature of this association may be complex, and the causal direction(s) uncertain. Guided by the conceptual overview and review of existing literature, we also highlight key areas for further research. The ultimate goal of this line of work is to elucidate mechanisms underlying any link between loneliness and accelerated aging in PTSD, and to develop, validate, and refine prevention and treatment efforts.

## 1. Introduction

People with Posttraumatic Stress Disorder (PTSD) are at higher risk for early or accelerated biological aging [1-3]. There are multiple ongoing efforts to elucidate the biological mechanisms of this association, including several components of allostatic load [4]. However, it is also critical to consider potentially modifiable factors at the social and clinical level. In the search for such factors, chronic loneliness may be a particularly strong candidate, yet has received little empirical attention to date [5]. Outside the context of PTSD, the “loneliness epidemic”, especially among older adults, has been increasingly noted internationally in the past few years from a wide diversity of researchers, clinical providers, and government policy-makers [6,7].

The topic of loneliness and social isolation have particular relevance to cognitive and health outcomes among older adults. In 2020 The National Academies of Sciences, Engineering, and Medicine published a comprehensive report on the problems of social isolation and loneliness among older adults, and its impact on health outcomes [8]. As noted within that report, up to 43% of adults age 60 years or above reported loneliness. Age-related factors may also influence the onset or maintenance of loneliness such as loss of spouses or partners, narrowing changes of one’s broader social network, declines in health and independent functioning as well as other environmental and psychological factors [9].

In their widely cited meta-analysis of 70 relevant studies of loneliness in the general population, Holt-Lunstad et al. [10] found that the all-cause mortality risk [Odds Ratio (OR)] of loneliness was equal to 1.49. Even after adjusting for potential confounds, the OR remained significant at 1.26. The latter risk of mortality is equivalent to that of grade 2 and 3 obesity (BMI ≥ 35), and is equivalent to smoking 10–14 cigarettes per day [11,12]. Loneliness has also been documented as a risk factor for cardiovascular disease, cognitive dysfunction and dementia, metabolic syndrome and type 2 diabetes, suicide, sleep disturbance, functional dependence, lower life-satisfaction, substance use, and, among the elderly, development of physical frailty [8]. Of note, this list of comorbidities overlaps strongly with those described in the premature senescence of PTSD [1]. This overlap of effects on health outcomes raises the questions as to the prevalence of loneliness among older and younger adults with PTSD, and the potential contribution of loneliness among such persons to accelerated biological aging.

We searched the empirical literature in order to determine the current state of knowledge regarding loneliness in PTSD, as well as its potential contribution to the accelerated aging associated with this disorder. In the sections below we first provide a conceptual overview of the potential relevance of loneliness to PTSD across the adult age range, and to the core symptom of “feelings of detachment or estrangement from others”. That overview is followed by a summary of findings from our review of relevant empirical literature. We close with recommendations for future research and clinical care.

## 2. Conceptual Relevance of Loneliness, Social Isolation, and Social Detachment to PTSD and Aging

*Loneliness* is commonly defined as a feeling of psychological distress resulting from a discrepancy between one’s desired social relationships (in terms of type, quality, and/or quantity), versus those relationships that one perceives having in their life [13]. Two key aspects of this definition are feelings of *distress* (rather than a neutral or desired state) and that the distress is grounded in a *perceived* deficit, regardless of the objective numerical size of one’s social network and available emotional and instrumental social support. These characteristics highlight a critical distinction between loneliness and objective social isolation, the latter which refers to an objectively quantifiable reduction or absence in the size or nature of an individual’s social environment such as frequency of social interactions and/or size of their social network [8]. Social isolation or solitude does not necessarily equate with loneliness, as it can be experienced as a positive state for some individuals or at certain times for many people [14-16].

Because loneliness can occur in the absence of objective social isolation, some investigators used the term *perceived social isolation*. Use of the term *perceived social isolation* is clearly on the rise in the empirical literature [17-28]. However, defining perceived social isolation as synonymous with loneliness may add inadvertent confusion given that, at its core, loneliness is a state of dysphoria and not simply a perception [16]. If focusing solely on the term *perception*, the key concept of *distressing feeling* may be inadvertently overlooked. This problem of identifying loneliness as a perception of social isolation permeates the literature and might be less of an issue if everyone assumed that the “perception of social isolation” inherently involves a dysphoric component. Unfortunately, this is not the case, and this confusion may have a substantial impact on interpretation of the criterion of “feelings of detachment from others” present in all editions of the DSM published since 1980.

Although there had been earlier descriptions of mental syndromes resulting from combat-related trauma, PTSD was not recognized as a formal diagnosis until the 1980 publication of the DSM-III [29]. As we have noted elsewhere [30], this delay in the formal recognition of this syndrome has had a particularly deleterious impact on the current cohort of older adults as the currently recognized evidence based trauma-focused therapies did not emerge until 1989 through 2001, many decades after some of the individuals in the older age cohort first experienced their index trauma. Since the 1980 publication of DSM-III, the symptom of feelings of detachment from others has remained a core part of the diagnostic criteria throughout the various editions up through the current DSM-5-TR [31] where it remains as Criterion D.6., “feelings of detachment or estrangement from others”. The stable presence of this criterion is particularly notable given other substantive changes to the diagnosis of PTSD over the years, including substantial changes in the types of traumatic experiences that meet Criterion A.

There are two important characteristics of the feelings of social detachment described in the DSM. First, it must be *associated with a psychological trauma*—i.e., it occurred or worsened after the traumatic event. Second, the social detachment or estrangement is not described in terms of objective social isolation, but rather as *feeling[s] of detachment*. In the case of objective social isolation, it is possible for individuals to isolate themselves by choice, unassociated with any feelings of discomfort or distress. Although it is possible to interpret the criterion in the DSM as related to loneliness, it is unclear whether this is true because feelings of detachment or estrangement may often, but not necessarily always, be distressful in nature.

## 3. Empirical Review

### 3.1. Literature Search

In order to determine what is presently known about loneliness in PTSD, in spring 2021 we conducted a search of the PubMed database using the following search string: ((“lonelier”[All Fields] OR “loneliness”[MeSH Terms] OR “loneliness”[All Fields]) AND (“stress disorders, post traumatic”[MeSH Terms] OR (“stress”[All Fields] AND “disorders”[All Fields] AND “post traumatic”[All Fields]) OR “post-traumatic stress disorders”[All Fields] OR “ptsd”[All Fields])) OR ((“lonelier”[All Fields] OR “loneliness”[MeSH Terms] OR “loneliness”[All Fields]) AND “post”[All Fields] AND (“traumatic”[All Fields] OR “traumatically”[All Fields] OR “traumatism”[All Fields] OR “traumatisms”[All Fields] OR “traumatization”[All Fields] OR “traumatizations”[All Fields] OR “traumatize”[All Fields] OR “traumatized”[All Fields] OR “traumatizes”[All Fields] OR “traumatizing”[All Fields]) AND (“stress”[All Fields] OR “stressed”[All Fields] OR “stresses”[All Fields] OR “stressful”[All Fields] OR “stressfulness”[All Fields] OR “stressing”[All Fields])) OR (“perceived social isolation”[All Fields] AND (“stress disorders, post traumatic”[MeSH Terms] OR (“stress”[All Fields] AND “disorders”[All Fields] AND “post traumatic”[All Fields]) OR “post-traumatic stress disorders”[All Fields] OR “ptsd”[All Fields])) OR (“perceived social isolation”[All Fields] AND (“post”[All Fields] AND (“traumatic”[All Fields] OR “traumatically”[All Fields] OR “traumatism”[All Fields] OR “traumatisms”[All Fields] OR “traumatization”[All Fields] OR “traumatizations”[All Fields] OR “traumatize”[All Fields] OR “traumatized”[All Fields] OR “traumatizes”[All Fields] OR “traumatizing”[All Fields]) AND (“stress”[All Fields] OR “stressed”[All Fields] OR “stresses”[All Fields] OR “stressful”[All Fields] OR “stressfulness”[All Fields] OR “stressing”[All Fields]))).

This search returned 140 reports published through 24 April 2021. We subsequently removed articles if loneliness was not mentioned in the abstract (n =16), if significant medical illnesses were associated with the traumatic event or events (n = 39), if the trauma was related to migration or forced removal from homeland (n = 11), if it was related to bereavement (n = 8), or if the content of the article revealed no information related to the thesis of this review (n = 35). This left 31 articles [32-62]. Careful reading of the retrieved articles also revealed citations to an additional 8 relevant articles that had not been identified through the search [63-70], bringing the total to 39 articles.

While preparing the review, we were aware of an increase in studies of loneliness related to the social distancing requirements during the first year of the global COVID-19 pandemic. On the possibility that this situation might have resulted in additional relevant publications focused on people with PTSD, we reran the original search criteria up through 8 July 2022. Because the initial search had resulted primarily in studies of post-traumatic stress symptoms (PTSS) rather than people diagnosed with PTSD, our key focus in the updated search was to identify any additional reports on the association of loneliness and social isolation with syndromal PTSD. This resulted in two additional studies [71,72], although there were numerous additional studies focused on PTSS.

### 3.2. Findings from Empirical Literature

We found no published epidemiologic reports on the prevalence of loneliness among people diagnosed with PTSD. With the exceptions described below, studies of loneliness among people formally diagnosed with PTSD are scarce. Two recent exceptions include a study reported by Sippel et al. [59] which found higher levels of loneliness and smaller social network sizes among 31 Veterans with DSM-5 diagnoses of PTSD, relative to 21 trauma exposed Veterans without PTSD. The second study, by Ypsilanti et al. [72], found that loneliness was significantly higher in Veterans with PTSD compared to people from the general population without PTSD. Unfortunately, the Sippel et al. study was limited to persons ages 18–55 years and the Ypsilanti et al. was limited to persons aged 20–66 years, so did not evaluate possible interactions with advanced age or accelerated aging per se. On the other hand, in a much earlier, albeit purely descriptive report, Macleod [46] described interviews with 45 World War II Veterans with DSM-III-R diagnoses of PTSD, ages 67–85 at the time of interview. They noted that among other factors such as declines in health and physical independence, loneliness “tended to be acknowledged” as a factor by the Veterans in aggravation of mental health (p. 628).

Another recent study that examined loneliness among people with syndromal PTSD was described in a recent report by Rutherford et al. [71]. These investigators compared trauma exposed persons over age 50 years with pre-pandemic chronic PTSD (n = 30) to individuals with no PTSD (n = 46) using assessments conducted early in the global COVID pandemic (April and May 2020). They found no significant group differences in levels of loneliness reported during follow-up assessment but the rates of what the authors described as “significant loneliness” were quite high in both groups (both approximately 63%). Unfortunately, the basis for the authors’ chosen cut-score for “significant loneliness” on their measure was unclear. [The absence of consensus for defining cut-scores on such scales has been a problem plaguing the overall loneliness research literature [5]].

In contrast to the limited number of reports on loneliness among persons with DSM diagnoses of PTSD, a number of studies have shown a significant correlation between loneliness and severity of post-traumatic stress symptoms (PTSS) (for example, see [34,36,40,45,56-58]). There is also evidence that loneliness significantly contributes to PTSS and loneliness may be a risk factor for later development of PTSD [40,60,61,73,74]. In one recent study, Cohn-Schwartz et al. [75] reported that older adults with a pre-morbid combination of what the investigators labeled “PTSD” and depressive symptoms were more likely to report increases in loneliness (and depression) during the pandemic. Taken together, these raise the possibility that the association may be bi-directional: loneliness may be a risk factor for development of PTSD in response to significant trauma, and PTSD may be a risk factor for subsequent social isolation and loneliness. Unfortunately, PTSD was not determined with a formal diagnostic interview, but rather via a cut-score on a symptom rating scale. Na et al. [76] found that Veterans with pre-pandemic PTSD symptom rating scales above a standard cut-off score were at increased risk for loneliness that persisted into the pandemic period, although this pattern was also present for Veterans with other mental health concerns such as depression and anxiety. Gonazlez-Sanguino et al. [77] reported older age, among other sociodemographic variables, to be inversely associated with PTSS (as well as depressive and anxiety symptoms) during the pandemic.

The laboratory of Solomon and colleagues has been the most active group in terms of studying loneliness and PTSS. In particular, they have found important associations of loneliness and PTSS in combat veterans and POWs from the 1982 Lebanon War [43,49-53,78]. Overall Solomon’s research group has found loneliness to be commonly associated with symptoms of PTSD, and that soldiers who received frontline treatment for post-traumatic stress demonstrated less loneliness over time. Importantly, these investigators found that loneliness in the context of posttraumatic stress has significant effects on psychosocial functioning, including marital adjustment, with loneliness serving as a significant mediator between PTSS and measures of subsequent marital adjustment [43]. These investigators have therefore highlighted the importance of addressing “a sense of isolation” during therapy. These investigators have further found that PTSS are not only associated with more severe loneliness, but also have a worsening effect on suicidal ideation. Loneliness, however, was not directly impacted by the type of trauma, nor was its relation to suicidal ideation linked to subsequent PTSS [79].

Although our search generally excluded PTSD related to -refugee status (due to the unique qualities of the criterion A traumas), it may be worth noting that there were interesting results reported in a study by Aragona et al. [80] from a sample of Chinese refugees who had fled religious persecution. They found “loneliness and boredom”, in addition to “Feeling that you do not know where you will lend up tomorrow” and “Not being able to find work”, were significantly related to the likelihood of having a PTSD (albeit defined by a rating scale) even when the threat and fear of tracking was no longer present. This evidence suggests that loneliness, along with the typical features associated with PTSD such as approach-avoidance, can add to one’s experience of emotional distress and burden following traumatic events, thereby exacerbating symptoms in a cyclical cycle.

The multiple studies discussing the association of PTSS and loneliness are limited in that the patterns of association may not generalize to people with actual syndromal PTSD. Van Zelst et al. [62] found higher loneliness among people with subsyndromal vs. full PTSD, although the sample with full PTSD was quite small (n = 11) and both groups had higher loneliness relative to the non-post-traumatic stress control subjects.

Dagan and Yager [37] suggested that loneliness may play a particular role in development and persistence of a debated syndrome called “Complex PTSD” [81,82]. In an analysis of survey data from a non-patient sample of older adults (ages 60 to 70), Fox et al. [70] found significant associations between loneliness and the symptoms of “disorganization of self-organization” thought to distinguish complex PTSD from standard PTSD. We found no other published empirical reports on loneliness among people with the Complex PTSD syndrome though this is clearly a dimension warranting further empirical attention. Unfortunately, we also found no studies investigating the effects of loneliness on persistence of a PTSD diagnosis.

We found no studies of the effects of standard PTSD interventions on loneliness and social disconnection among persons diagnosed with PTSD. However, Felton et al. [38] conducted an interesting study of Interpersonal Therapy (IPT) on loneliness and PTSS among 181 incarcerated persons with major depressive disorder and a history of trauma. They found both loneliness and PTSS were significantly reduced after the IPT intervention. However, contrary to expectation, hopelessness and depressive symptoms were found to underlie the relationship between treatment intervention and PTSS rather than through changes in perceived social support or loneliness. It is unclear why loneliness did not mediate the relationship between IPT and PTSS as this intervention would presumably address interpersonal difficulties thereby reducing loneliness. However, it should be noted that IPT is not typically considered a gold-standard treatment for PTSD compared to other established interventions such as prolonged exposure (PE), cognitive processing therapy (CPT), or eye movement desensitization and reprocessing (EMDR). Further randomized controlled trials that include loneliness in PTSD intervention studies could help elucidate any role of loneliness in reducing PTSS.

## 4. Discussion

Both loneliness and PTSD have been reported to have deleterious effects on biological aging and other public health consequences. As reviewed above, there is currently a sparse amount of empirical research specifically focused on disentangling the relationships between loneliness, social isolation, and feelings of detachment among persons with PTSD, regardless of whether focused on older adults or the question of accelerated aging in PTSD. However, the growing literature on the association of loneliness with post-traumatic stress symptoms (PTSS) suggest there may be an increase of loneliness among people with this disorder; the potential role that loneliness may play in accelerated aging warrants thorough empirical research.

A few limitations of this report should be noted. Foremost, the nature of our literature search did not facilitate completion of a definitive PRISMA flow-chart as our initial search up through early 2021 (including studies of PTSS) were later supplemented with a July 2022 search to identify studies that were specifically focused on people with PTSD. The original search had already provided sufficient evidence for an association of PTSS with loneliness, but for reasons described above those may not generalize to syndromal PTSD. Much of the research published in the wake of the global COVID-19 pandemic social distancing requirements was survey based and, therefore, PTSS-related. We recognize that the changed nature of the search toward later identification of solely PTSD studies in the second (July 2022) updated of the review hampers direct replication but have included the search terms above to foster such efforts. A second potential limitation is that we did not limit the review to studies focused on aging or accelerated aging. However, this was intentional given the need to first establish whether loneliness is a clear issue within PTSD broadly.

One limitation in the literature reviewed above, and the broader empirical literature on loneliness in other populations, is the lack of consistency in measurement of loneliness. Some of the most common measures in the broader literature, and similar to that on loneliness in PTSD, include the 20-item UCLA Loneliness Scale (in its original 1978 form [83], the 1980 revised version [84], or the 1996 third edition [85]), the 11- or 6-item versions of the De Jong-Gierveld Loneliness Scale [86,87], and the 3-item Hughes et al. [88] abbreviation of the version of the 1980 UCLA scale. Other common choices are single item-measures, such as item 14 from the Center for Epidemiologic Studies–Depression Scale (CESD) [89]. Single-item measures tend to ask directly about frequency of loneliness (e.g., CESD item 14 asks respondents to report the frequency of loneliness over the preceding week in response to the statement “I felt lonely”). In contrast, items on the UCLA scales intentionally avoided use of the word “lonely” due to concerns about under-reporting due to perceived stigma. Thus, different methods may lead to different rates of clinically relevant loneliness [90]. It may also be noted that most existing measures provide information about severity of loneliness but not about the phenomenological experience of loneliness in regard to the quality of the psychological distress, such as sadness, anger, or anxiety [9].

At present any conclusions about the role of loneliness in accelerated aging in PTSD are mostly speculative, but we here draw some observations in the hope of stimulating further research, discussion, and development in this area. Both loneliness and PTSD have been reported to increase mortality. At an organ system level there is strong evidence for increased cardiovascular disease and morbidity, as well as metabolic morbidity including high LDL cholesterol, triglycerides, and reduced HDL as well as type 2 diabetes in loneliness [91-94] and PTSD [95-100]. Both conditions have also been reported to be associated with impaired cognitive functioning [1,101-106].

At a deeper physiological level, inflammatory processes may provide a logical link between loneliness and accelerated aging in PTSD. Elevated inflammatory indices such C-reactive protein (CRP), interleukin 6 (IL-6), and tumor necrosis factor alpha (TNFα), have been found associated with loneliness [107-110] and with PTSD [111-126]. The same is true of elevated indices of oxidative damage [127-129]. Inflammatory cytokines can be actively imported into the central nervous system (CNS), and can heighten brain inflammation [130]. In the brain, pro-inflammatory cytokines may have not only neurotoxic effects but also effects on synaptic transmission [131], which could provide a theoretical bridge between psychiatric symptoms and the general aging process. Thus, inflammatory processes provide a logical link between loneliness, PTSD symptoms, and age-related morbidity [96,132,133].

Apart from physiological overlap, another major model that may relate loneliness and PTSD has included indirect, behavioral, and social/environmental links between loneliness and health. This “social control” model posits that close healthy relationships discourage negative health behaviors while promoting positive health behaviors, and are also associated with health-promoting environmental resources, such as good transportation, nutritious food, and ease of healthcare access [134-136].

Because of the importance of both social and physiological factors, Cacioppo and colleagues proposed a widely cited integrated model of loneliness, combining both the social aspects and the pathophysiological evidence [137-139]. Their model has several components relevant to the initiation and maintenance of loneliness and its social, social-cognitive, and biological effects. However, with regard to the direct physiologic mechanisms, they posit that persistent/chronic loneliness is associated with sustained physiologic stress responses, in which there is chronic overdrive of the sympathetic nervous system which may result in dysregulation of inflammatory and endocrine responses, thus elevating risk for medical comorbidity. This model has obvious relevance to PTSD given that sustained stress responses are also a key feature [4].

All of this suggests an important testable hypothesis: PTSD may exert its biological aging and other public health effects to a large extent through loneliness. Loneliness has been found to be a mediator of other conditions such as paranoia and other forms of adult psychopathology [140,141]. Because of the paucity of literature on loneliness in PTSD at this time, it is impossible to gauge how likely it is that loneliness is a mediator or even a moderator of PTSD, but considering the overlap in morbidity and pathophysiological findings between the two, it must be taken seriously as a possibility.

## 5. Summary and Conclusions

Although the present state of the empirical literature is such that definitive conclusions should be avoided, there is sufficient reason to suspect a link between loneliness and PTSD, as well as that that link could potentially contribute to the accelerated biological aging associated with this disorder. Perceived social estrangement or isolation from others has been seen as one of the core symptoms of PTSD since the inception of the diagnosis in the 1980 DSM-III, and through all subsequent versions through the current DSM-5-TR. Unfortunately, it appears that loneliness has been neglected in most studies of PTSD; we suggest two possible reasons for this. First, some investigators may assume that loneliness is already addressed in PTSD because of loneliness being equated with perceived social isolation, and that is already covered in the DSM. Second, others may assume that because the social isolation may be self-imposed and therefore associated more with avoidance, that it then would not be necessarily a loneliness-related phenomenon.

Nevertheless, findings from the limited number of existing relevant empirical reports suggest that loneliness is associated with PTSD, but that the relationship may be complex. Of note, research in other mental health populations such as depression and anxiety raise the possibility of bidirectional relationships between psychiatric symptoms and loneliness [5]. Importantly, loneliness is not only an aversive experience that merits investigation for that reason alone, it is also associated with high medical comorbidity, cognitive decline, and early mortality. The mechanisms by which loneliness affects physical and cognitive health are presently unknown, but there is compelling data suggesting at least partial mediation by physiologic dysfunction. There is substantial overlap between the types of medical and cognitive morbidity associated with loneliness and those associated with PTSD, as well as with the candidate pathophysiological mechanisms involving inflammatory dysfunction and metabolic changes.

Although, as we hope to have shown, there is a strong case for the potential importance of loneliness in PTSD, very little is known about critical *epidemiological* variables such as incidence or prevalence of loneliness in this population. There is even less information about other characteristics of loneliness in PTSD, such as its relationship to demographic variables including age, gender, socioeconomic status, and many others. The situation of aging with PTSD is particularly complex given age-related changes in memory and unknown long-term effects of untreated PTSD.

The DSM criteria for PTSD include “feelings” of social isolation, but, as we noted, this is not necessarily the same as loneliness. Thus, the *subjective experience of loneliness* and in particular its dysphoric nature and its relationship to objective social isolation in PTSD requires further study. Studies to date have failed to look closely at item-level analysis, such as factor analysis comparisons, between how people with PTSD respond to specific items on loneliness self-report assessments. Careful investigation at the item level would provide much added clarity regarding the associated links, as well as novel factors, between loneliness and PTSD. Additionally, qualitative research is critical to explore the nature of loneliness from the perspective of those afflicted. This is particularly important when considering the nature of risk factors. This also relates to the question of whether loneliness itself should be included in the diagnostic criteria for PTSD.

With the potentially complex relationship of loneliness and PTSD (whereby PTSD may be a risk factor for loneliness, but loneliness may also be a risk factor for the re-emergence of PTSD later in life), the *nature of the causal relationships* requires further investigation and clarification.

Of note, for *both* PTSD and loneliness, there is evidence for aberrant physiological mechanism involving inflammatory, cardiovascular, and metabolic systems. This raises the question of what the *importance and relationship of the pathophysiology of loneliness* is to accelerated biological aging in PTSD. For example, does loneliness exacerbate the physiological abnormalities already a part of PTSD, or is loneliness a potential mediator of some of the findings observed in PTSD? This may also have impact on aging, which shares many of these physiological features with both PTSD and loneliness [1]. The reports of loneliness impacting *cognitive functioning and dementia* are also important to follow up in terms of studies of PTSD where impairment in cognitive functioning has also been reported. Given the possible deleterious impact of loneliness, PTSD, and age, it is imperative to continue to evaluate to what extent their combined influences impact cognitive and physical health in older adults, particularly through prospective, longitudinal studies.

Finally, there are several areas worthy of investigation relating to treatment and intervention. It is important to determine whether standard evidence-based treatments for PTSD such as CPT or PE have an impact on loneliness and/or whether loneliness has a potentially moderating effect on the relative effectiveness of these treatments. There is preliminary evidence for the reduction of loneliness among people provided with complementary psychosocial interventions (reviewed above); however, the mechanisms by which these effects are achieved also warrant systematic investigation. Another primary target for future study includes evaluating the potential for cognitive and physical health improvements following loneliness and PTSD interventions in older adults. Ideally, a multi-armed approach with treatment as usual, PTSD-only treatment, and PTSD+loneliness treatment would help elucidate the potential synergistic impact of loneliness and PTSD intervention on aging.

## Abbreviations

BMI: Body Mass Index
CESD: Center for Epidemiologic Studies—Depression Scale
CNS: central nervous system
COVID: corona virus disease
CPT: cognitive processing therapy
CRP: C-reactive protein
DSM: Diagnostic and Statistical Manual
DSM-III: Diagnostic and Statistical Manual—Third Edition
DSM-5-TR: Diagnostic and Statistical Manual—Fifth Edition—Text Revision
EMDR: eye movement desensitization and reprocessing
HDL: high density lipoprotein
IL-6: interleukin 6
IPT: interpersonal therapy
LDL: low density lipoprotein
OR: odds ratio
PE: prolonged exposure
PTSD: posttraumatic stress disorder
PTSS: posttraumatic stress symptoms
TNFα: tumor necrosis factor alpha
UCLA: University of California Los Angeles
